# Supplementary material for: Characterizing Powdered Activated Carbon Treatment of Surface Water Samples Using Polarity-Extended Non-Target Screening Analysis
Source: Molecules. 2022 Aug 16;27(16):5214. doi: 10.3390/molecules27165214 (PMC9415745; doi:10.3390/molecules27165214)
Supplement: Supplementary file 1 [file molecules-27-05214-s001.zip › molecules-1852489-supplementary.pdf]

Supplementary material of “Characterizing powdered activated carbon treatment of surface water samples using polarity-extended non-target screening analysis”

Susanne Minkus [1, 2], Stefan Bieber [2] and Thomas Letzel [1,2,*]

1 Technical University of Munich (Chair of Urban Water Systems Engineering), Garching, Germany
2 Analytisches Forschungsinstitut für Non-Target Screening (AFIN-TS) GmbH
* Correspondence: t.letzel@afin-ts.de

Table S1: Means and standard deviations of $\log_2$(fc) values for the internal standards and the polar standard compounds measured in negative ionization mode. H118, H120 and H121 are the laboratory names of the different PAC types (Table 1) which were tested for surface water treatment at three different concentrations.

	**H118**	**H120**	**H121**
**Internal standards**	n = 10	n = 10	n = 10
2 mg L^{-1}	0.07 ± 0.05	-0.24 ± 0.05	0.06 ± 0.07
7 mg L^{-1}	0.14 ± 0.09	-0.32 ± 0.08	0.18 ± 0.07
30 mg L^{-1}	0.04 ± 0.08	-0.37 ± 0.07	0.23 ± 0.07
**Polar standards**	n = 2	n = 3	n = 5
2 mg L^{-1}	-0.13 ± 0.12	-0.12 ± 0.24	-0.35 ± 1.16
7 mg L^{-1}	0.20 ± 0.73	-0.25 ± 0.37	-2.16 ± 3.04
30 mg L^{-1}	0.18 ± 0.86	-0.75 ± 1.19	-1.59 ± 2.94

Table S2: Means and standard deviations of $\log_2$(fc) values for the non-target features in negative ionization mode. H118, H120 and H121 are the laboratory names of the different PAC types (Table 1) which were tested for surface water treatment at three different concentrations.

	**Number of features**	**Mean $\log_2$(fc)**	**Increasing/decreasing features [%]**	**Significant feature**
**H118**				
2 mg L^{-1}	2318	-0.02 ± 0.31	0.3/0.5	0
7 mg L^{-1}	2433	-0.13 ± 0.51	1.6/4.5	121
30 mg L^{-1}	2482	-0.05 ± 0.55	2.6/3.7	118
**H120**				
2 mg L^{-1}	2378	0.19 ± 0.34	2.9/0.0	17
7 mg L^{-1}	2332	0.21 ± 0.36	3.5/0.2	50
30 mg L^{-1}	2333	0.30 ± 0.38	6.7/0.2	125
**H121**				
2 mg L^{-1}	2277	-0.03 ± 0.35	0.2/0.7	0
7 mg L^{-1}	2357	-0.10 ± 0.37	0.2/1.6	0
30 mg L^{-1}	2402	-0.06 ± 0.38	0.1/1.3	4

Table S3: Table of internal standards spiked into samples after treatment and prior to LC-MS analysis

Name	InChIKey	Chemical formula	Log D (pH 7)	Solvent stock	C(Stock) [µM]	Manufacturer
**6-amino-1,3-dimethyl-5-(formylamino) uracil**	ZNDGAXCBZGSJGU-UHFFFAOYSA-N	C7H10N4O3	-2.00	ACN/H2O (50/50)	1000	Sigma
**Etilefrine**	SQVIAVUSQAWMKL-UHFFFAOYSA-N	C10H15NO2	-1.42	ACN	1000	Sigma
**Sotalol**	ZBMZVLHSJCTVON-UHFFFAOYSA-N	C12H20N2O3S	-2.47	ACN	586	Sigma
**Vidarabine**	OIRDTQYFTABQOQ-UHTZMRCNSA-N	C10H15N5O5	-2.1	ACN/H2O (50/50)	337	
**Chloridazon**	WYKYKTKDBLFHCY-UHFFFAOYSA-N	C10H8ClN3O	1.11	ACN/H2O (50/50)	1000	Sigma
**Chlorbromuron**	NLYNUTMZTCLNOO-UHFFFAOYSA-N	C9H10BrClN2O2	2.85	Methanol	1000	Dr. Ehrenstorfer
**Chlortoluron**	JXCGFZXSOMJFOA-UHFFFAOYSA-N	C10H13ClN2O	2.44	ACN	1000	Sigma
**Metconazole**	XWPZUHJBOLQNMN-UHFFFAOYSA-N	C17H22ClN3O	3.59	Methanol	1000	Sigma
**Metobromuron**	WLFDQEVORAMCIM-UHFFFAOYSA-N	C9H11BrN2O2	2.24	ACN	1096	Sigma
**Monuron**	BMLIZLVNXIYGCK-UHFFFAOYSA-N	C9H11ClN2O	1.93	Methanol	970	Sigma

Table S4: Polar standard compounds spiked into samples prior to PAC treatment.

Name	InChIKey	Chemical formula	Log D (pH 7)
**1,3-Dimethyl-2-imidazolidinone**	CYSGHNMQYZDMIA-UHFFFAOYSA-N	C5H10N2O	-0.64
**2,2,6,6-tetramethyl-4-piperidone**	JWUXJYZVKZKLTJ-UHFFFAOYSA-N	C9H17NO	-0.32
**2,4-diamino-6-(hydroxymethyl)pteridine**	CYNARAWTVHQHDI-UHFFFAOYSA-N	C7H8N6O	-1.37
**2-aminopyridine**	ICSNLGPSRYBMBD-UHFFFAOYSA-N	C5H6N2	0.30

**3-pyridinemethanol**	MVQVNTPHUGQQHK-UHFFFAOYSA-N	C6H7NO	-0.01
**Ectoine**	WQXNXVUDBPYKBA-UHFFFAOYSA-N	C6H10N2O2	-2.53
**Famotidine**	XUFQPHANEAPEMJ-UHFFFAOYSA-N	C8H15N7O2S3	-3.04
**4-(2-hydroxyethyl)morpholine**	KKFDCBRMNNSAAW-UHFFFAOYSA-N	C6H13NO2	-1.12
**Miglitol**	IBAQFPQHRJAVAV-ULAWRXDQSA-N	C8H17NO5	-3.89
**N,N'-ethylenebisacetamide; (N,N'-ethylenedi(diacetamide))**	WNYIBZHOMJZDKN-UHFFFAOYSA-N	C6H12N2O2	-1.78
**Acamprosate**	AFCGFAGUEYAMAO-UHFFFAOYSA-N	C5H11NO4S	-4.10
**L-Leucine**	ROHFNLRQFUQHCH-UHFFFAOYSA-N	C6H13NO2	-1.59

Table S5: Parameter settings of each processing step of the non-target screening workflow for comparative analysis of an untreated and a treated sample. Parameters that were sufficiently optimized in a previous study [1] are highlighted in green. Parameters that were adapted to the circumstances of the present investigation are marked in red. In case there was no need to optimize default settings they are depicted in blue. If parameter settings differed for negative ionization mode, the values are given in brackets.

Processing step	Description	Parameter	Setting	Comment
Mass detection		Algorithm	Wavelet transform	Detects peaks using continuous wavelet transformation using “Mexican Hat” wavelet
		Noise level	10,000	Minimum intensity of a data point to be considered in chromatogram
		Scale level	6	Stretches or compresses the wavelet
		Wavelet window size	30 %	Window size used to calculate wavelet
		RT range	5 min – 33 min	Corresponds to the HILIC and RPLC elution intervals
		MS levels	1+2	
		Polarity	+(-)	
		Spectrum type	Profile	Adapted to the raw data format of the vendor (Thermo Fisher Scientific)
Chromatogram building		Minimum group size in number of scans	5	

	Constructs EICs using the ADAP algorithms[2]	Group intensity threshold	20,000	Optimized to minimize total number of features and processing time and maximize recall of standard compounds (n=20)
		Minimum highest intensity	50,000	Optimized to minimize total number of features and processing time and maximize recall of standard compounds (n=20)
		m/z tolerance	0.0012 Da	Derived from targeted analysis: Maximum m/z span over all sample injections (n=35) and standard compounds (n=20), rounded up to 4th decimal
Smoothing	Applies Savitzky-Golay filter to	Filter width	25	Adapted to noisier data
Chromatogram deconvolution	Separates each chromatogram into individual peaks	Algorithm	Wavelets (ADAP)	
		S/N threshold	10	
		Minimum feature height	100,000 (50,000)	Optimized to minimize total number of features and processing time and maximize recall of standard compounds (n=20)
		Coefficient/area threshold	30	
		Peak duration range (low)	0.13 min	
		Peak duration range (high)	8.00 min	Adapted to achieve full recall of standard compounds (n=20)
		RT wavelet range (low)	0.03 min	
		RT wavelet range (high)	2.00 min	Adapted to achieve full recall of standard compounds (n=20)
		m/z center calculation	Median	
Peak filter	Eliminates peaks which do not meet the specified criteria	Number of data points	2 – 1600	Derived from targeted analysis: Minimum and maximum of all sample injections (n=35(36)) and standard compounds (n=20), rounded up and down, respectively.
		Tailing factor	0.40 – 8.30	
		Asymmetry factor	0.05 – 13.59	
Isotope grouping and removal	Recognizes isotopic patterns within defined RT and m/z ranges and removes all	m/z tolerance	0.0012 Da	Derived from targeted analysis: Maximum m/z span over all sample injections (n=35) and standard compounds (n=20), rounded up to 4th decimal

	peaks except the highest isotope	RT tolerance	0.62 min	Derived from targeted analysis: Maximum RT span over replicate injections and standard compounds (n=20), rounded up to 2nd decimal
		Maximum charge	1	
		Representative isotope	Lowest m/z	
Adduct tagging and removal	Recognizes adduct peaks within defined RT and mass range	RT tolerance	0.62 min	Derived from targeted analysis: Maximum RT span over replicate injections and standard compounds (n=20), rounded up to 2nd decimal
		Adduct m/z differences	21.9825 Da, 37.9559 Da, 17.0265 Da	
		m/z tolerance	0.0012 Da	Derived from targeted analysis: Maximum m/z span over all sample injections (n=35) and standard compounds (n=20), rounded up to 4th decimal
		Maximum relative adduct peak height	100 %	
Intra-sample alignment	Aligns peaks across technical replicates and corrects RT deviations based on RANSAC algorithm and non-linear regression model[3]	m/z tolerance	0.0015 Da	Adapted to achieve full recall of standard compounds (n=20)
		RT tolerance	1.00 min	Sets RT range to create the model for RT correction
		RT tolerance after correction	0.62 min	Derived from targeted analysis: Maximum RT span over replicate injections and standard compounds (n=20), rounded up to 2nd decimal
		RANSAC iterations	2000	Maximum number of iterations to find model
		Minimum number of points	20 %	Minimum portion of points required for a valid model
		Threshold value	0.07 min	Threshold for a data point to fit the model
Duplicate filter	Finds features of which the m/z and RT difference is lower than the predefined tolerances	Filter mode	New average	Creates consensus feature from duplicates
		m/z tolerance	0.0015 Da	Adapted to achieve full recall of standard compounds (n=20)
		RT tolerance	0.62 min	Derived from targeted analysis: Maximum RT span over replicate injections and standard compounds (n=20), rounded up to 2nd decimal
Replicate filter		Minimum peaks	3	
Inter-sample alignment	Aligns peaks across treated	m/z tolerance	0.0015 Da	Adapted to achieve full recall of standard compounds (n=20)

	and untreated sample and corrects RT deviations based on RANSAC algorithm and non-linear	RT tolerance	1.00 min	Sets RT range to create the model for RT correction
		RT tolerance after correction	0.62 min	Derived from targeted analysis: Maximum RT span over replicate injections and standard compounds (n=20), rounded up to 2nd decimal
		RANSAC iterations	2000	Maximum number to find model
		Minimum number of points	20 %	Minimum portion of points required for a valid model
		Threshold value	0.07 min	Threshold for a data point to fit the model
Gap filling	Searches for missing peaks using the m/z and RT range defined by the rest of the aligned peaks	m/z tolerance	0.0015 Da	Adapted to achieve full recall of standard compounds (n=20) and added to the m/z range constituted by the other peaks of the feature
Replicate filter		Minimum peaks	6	
Intensity normalization	The peak heights of a feature are normalized using internal standards	Normalization type	Weighted contribution of all standards	
		Peak measurement type	Peak height	
		Standard compounds	Internal standards	N = 10, compare Table S1

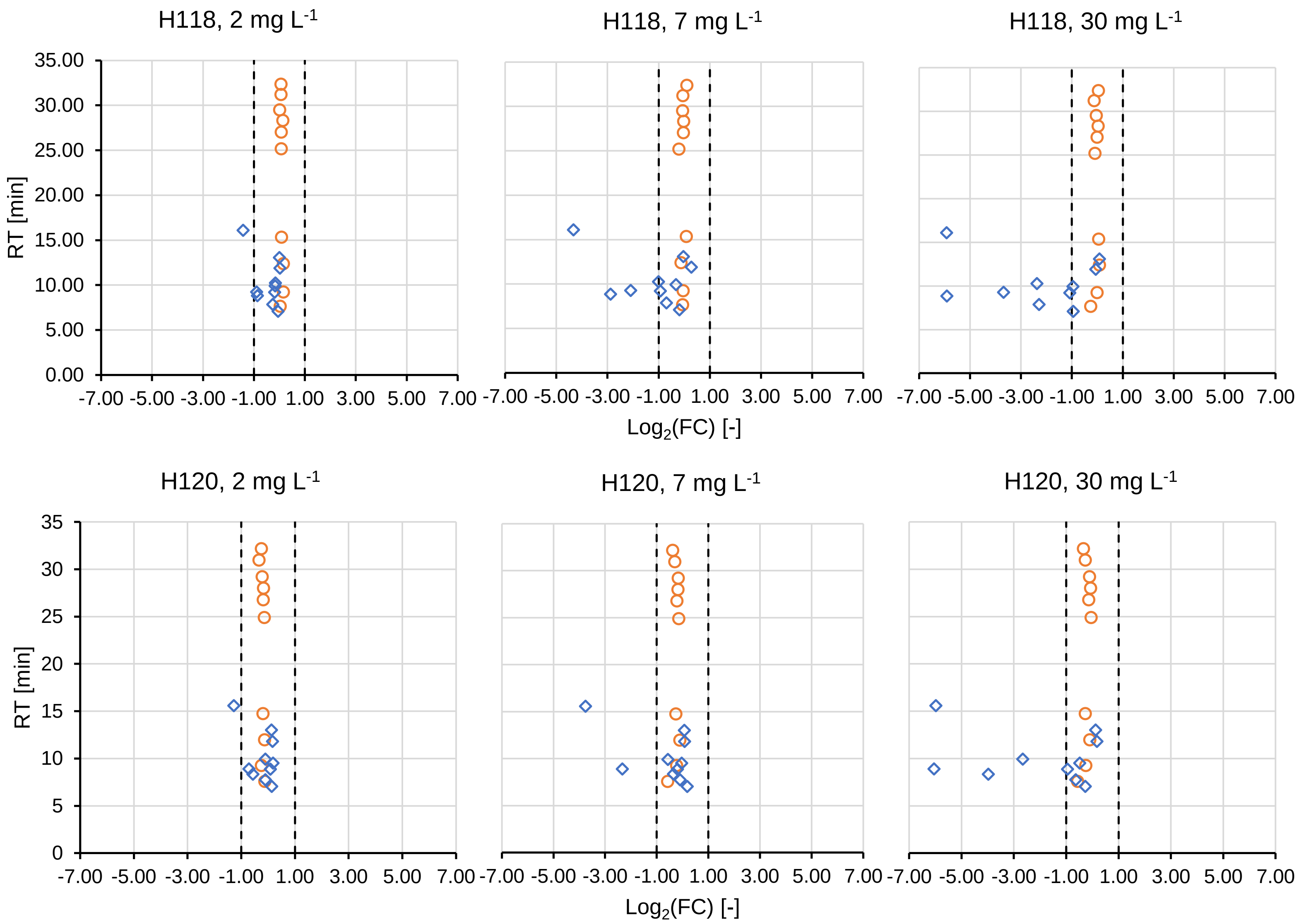

Figure S1: The base-2 logarithm of the fold changes of the polar standard compounds (blue diamonds) and the internal standards (orange circles) are plotted versus their retention times. The dashed lines mark the consistency interval where no compound removal is assumed. The data was recorded in positive ionization mode. The plots indicate that PAC H118 adsorbed the compounds famotidine, 2,4-diamino-6-(hydroxymethyl)pteridine, 3-pyridinemethanol, 2-aminopyridine and 4-(2-hydroxyethyl)morpholine. No decrease was observed for 4-(2-hydroxyethyl)morpholine when treating the sample with PAC H120.

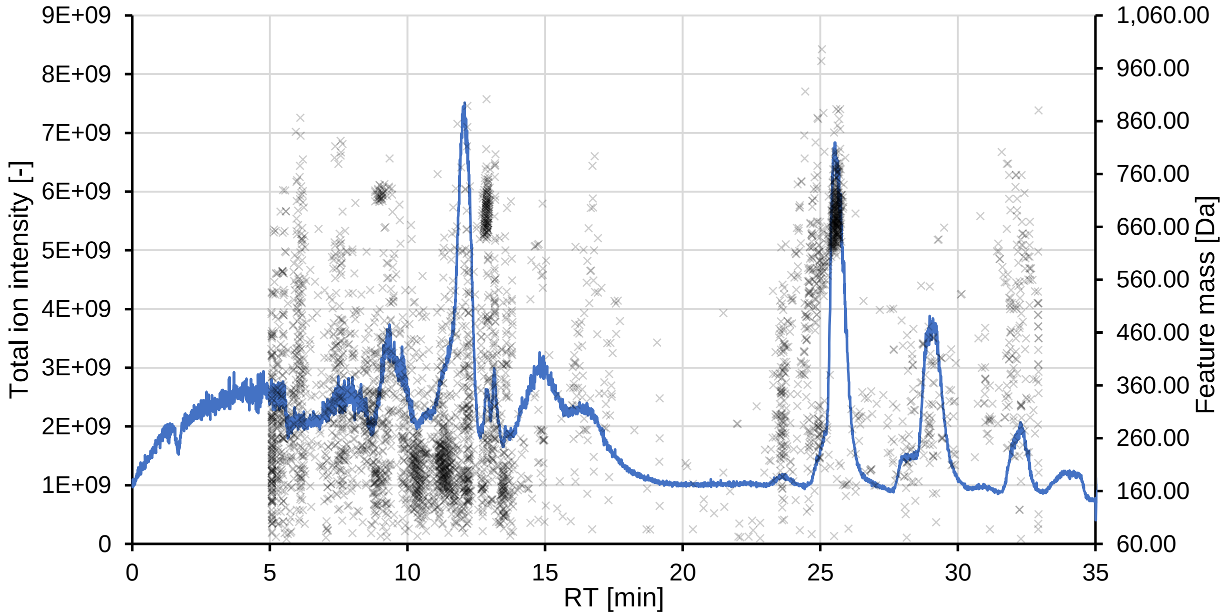

Figure S2: The total ion chromatograms (blue line, primary y-axis) and the non-target peaks (black crosses, secondary y-axis) are exemplarily displayed for the sample treated with the H118 PAC at 30mg L^{-1}, third replicate.

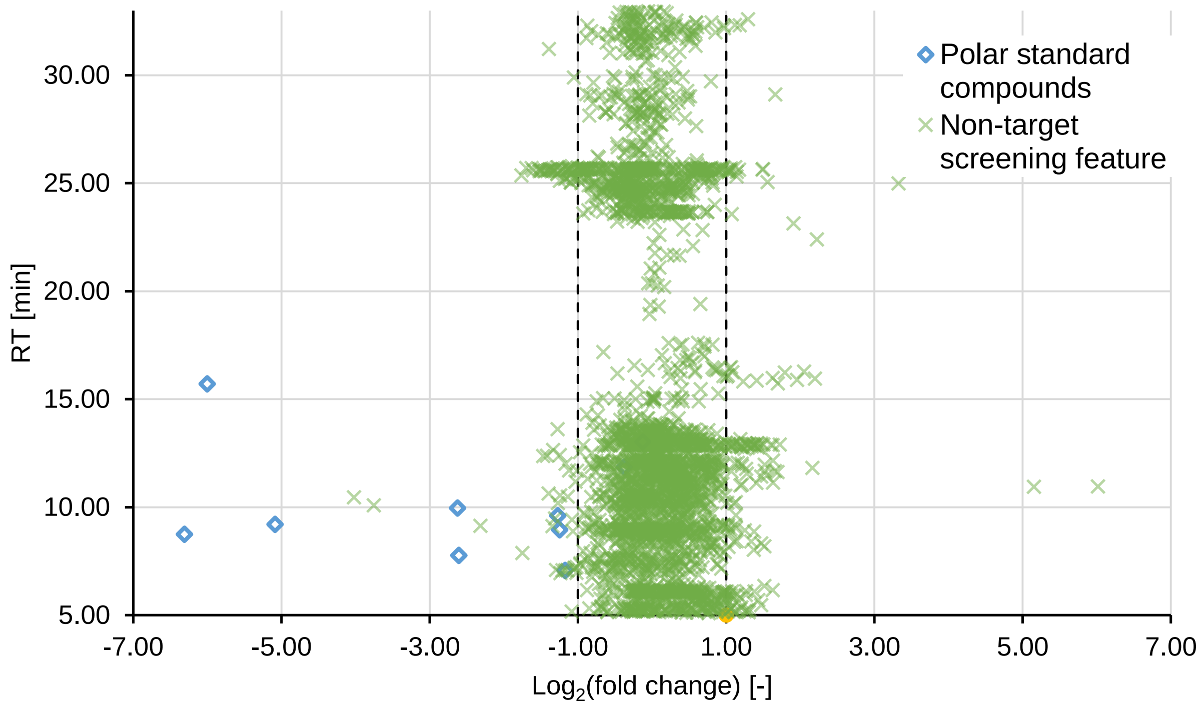

Figure S3: Non-target features (green crosses) and polar standard compounds (blue diamonds). The dashed lines mark the consistency interval. Log_2(fc) values < -1 and > 1 are defined as a decrease and increase in signal intensity, respectively. Here, the sample treated with 30 mg L^{-1} of PAC H118 was compared to the untreated blank sample, both measured in positive ionization mode.

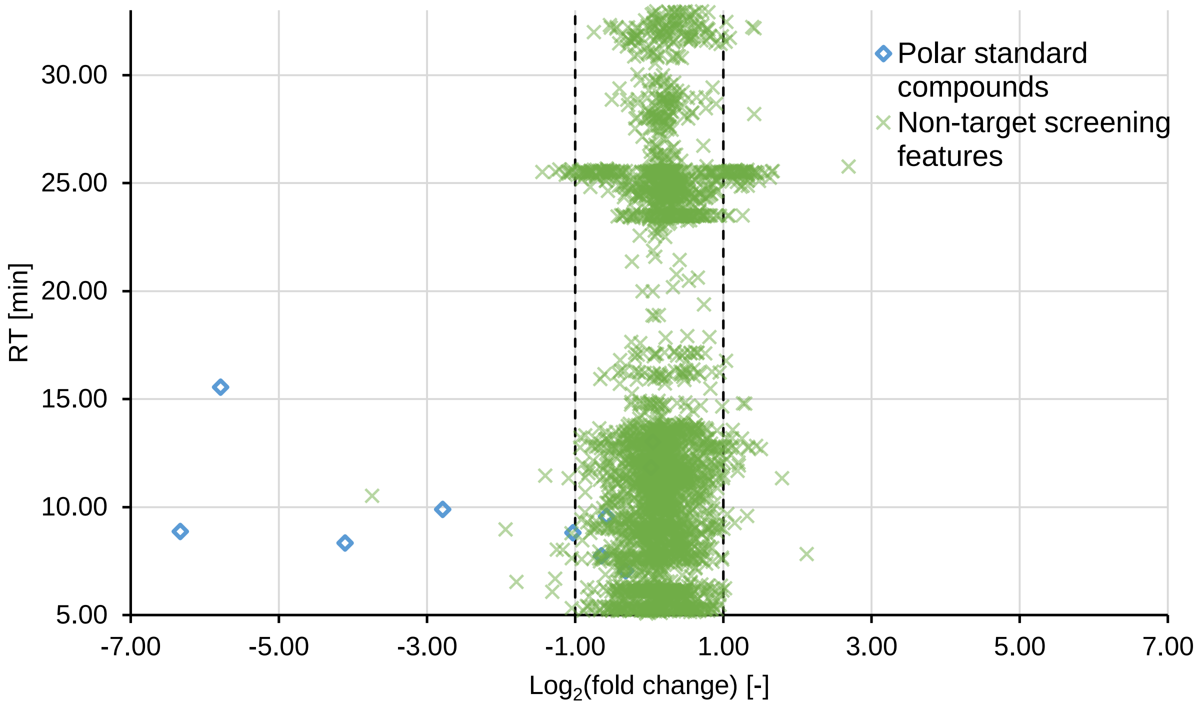

Figure S4: Non-target features (green crosses) and polar standard compounds (blue diamonds) of the sample treated with 30 mg L^{-1} of PAC H120 compared to the untreated blank sample, both measured in positive ionization mode.

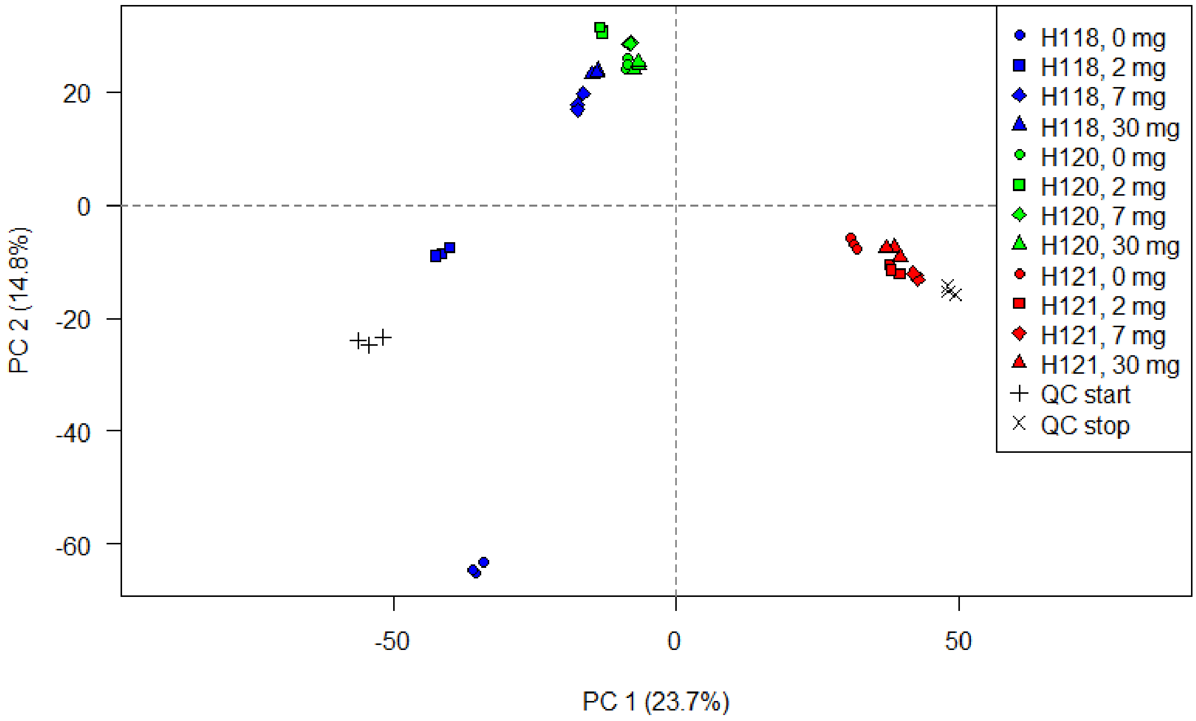

Figure S5: Scores plot of the PCA based on the normalized peak heights of the features extracted from each individual measurement in negative ionization mode.

References:

1. Minkus S, Bieber S, Letzel T (2021) (Very) polar organic compounds in the Danube river basin: Non-target screening workflow and prioritization strategy for extracting highly confident features. Anal Methods 13:2044–2054.https://doi.org/10.1039/D1AY00434D

2. Myers OD, Sumner SJ, Li S, Barnes S, Du X (2017) One Step Forward for Reducing False Positive and False Negative Compound Identifications from Mass Spectrometry Metabolomics Data: New Algorithms for Constructing Extracted Ion Chromatograms and Detecting Chromatographic Peaks. Anal Chem 89:8696–8703.https://doi.org/10.1021/acs.analchem.7b00947

3. Pluskal T, Castillo S, Villar-Briones A, Orešič M (2010) MZmine 2: Modular framework for processing, visualizing, and analyzing mass spectrometry-based molecular profile data. BMC Bioinformatics 11:https://doi.org/10.1186/1471-2105-11-395
